# What Produces Death

**Published:** 1889-12

**Authors:** 


### WHAT PRODUCES DEATH.

Some one says that few men die of age. Almost all persons die of disappointment, personal, mental, or bodily toil, or accident. The passions kill men sometimes even suddenly. The common expression, "choked with passion," has little exaggeration in it, for even though not suddenly fatal, strong passions shorten life. Strong-bodied men often die young—weak men live longer than the strong, for the strong use their strength, and the weak have none to use. The latter take care of themselves, the former do not. As it is with the body, so it is with the mind and temper. The strong are apt to break, or, like the candle, run; the weak burn out. The inferior animals, which live temperate lives, have generally their prescribed term of years.

The horse lives twenty-five years, the ox fifteen or twenty, the lion about twenty, the hog ten or twelve, the rabbit eight, the guineapig six or seven. The numbers all bear proportion to the time the animal takes to grow its full size. But man, of all animals, is one that seldom comes up to the average. He ought to live a hundred years, according to the physiological law, for five times twenty are one hundred; but instead of that, he scarcely reaches an average of four times the growing period. The reason is obvious—man is not only the most irregular and most intemperate, but the most laborious and hard-working of all animals. He is always the most irritable of all animals, and there is reason to believe, though we cannot tell what an animal secretly feels, that, more than any other animal, man cherishes wrath to keep it warm, and consumes himself with the fire of his own reflections.—*Scientific American.*

